# Association of Insurance Status and Extent of Organ Involvement With Survival Among Patients With Stage IV Cancer

**DOI:** 10.1001/jamanetworkopen.2022.17581

**Published:** 2022-06-17

**Authors:** Peter L. Zhan, Maureen E. Canavan, Theresa Ermer, Matthew D. Pichert, Andrew X. Li, Richard C. Maduka, Daniel J. Boffa

**Affiliations:** 1 Division of Thoracic Surgery, Department of Surgery, Yale University School of Medicine, New Haven, Connecticut; 2 Cancer Outcomes Public Policy and Effectiveness Research Center, Department of Internal Medicine, Yale University School of Medicine, New Haven, Connecticut

## Abstract

This cohort study examines the association of insurance status and extent of metastatic organ involvement with survival among patients with stage IV cancer to better understand outcome disparities in the US.

## Introduction

Insurance status has been identified as a factor associated with cancer outcomes, but this association has not been thoroughly examined among patients with stage IV cancer. Increasing metastatic burden is a negative prognostic factor in stage IV cancer. We examined the associations of insurance status and extent of metastatic organ involvement with survival among patients with stage IV cancer to better understand outcome disparities in the US.

## Methods

This retrospective cohort study used the National Cancer Database to identify patients aged 18 years or older who received a diagnosis of stage IV non–small cell lung, breast, pancreas, colon, and prostate cancer between 2016 and 2018. The Strengthening the Reporting of Observational Studies in Epidemiology (STROBE) reporting guidelines were followed. This study was performed in accordance with our institutional review board–approved protocol, with consent waived because the data were anonymous and publicly available, in accordance with 45 CFR §46. Violations in the Cox proportional-hazards assumption were detected via Schoenfeld residuals; therefore, multivariable time-to-event models incorporating sociodemographic variables and pattern of metastatic organ involvement were created to evaluate factors associated with survival. The association between socioeconomic characteristics and extent of metastatic involvement was assessed using multivariable logistic regression models incorporating socioeconomic variables (Table). Significance was set at 2-sided *P* < .05. Data were analyzed with SAS statistical software version 9.4 (SAS Institute). Data were analyzed from February to April 2022.

**Table.** Sociodemographic Characteristics of Patients With Stage IV Cancer and Multiorgan Metastatic Involvement at Diagnosis^a^

Characteristic	NSCLC (n = 113 756)		Breast (n = 23 862)		Pancreas (n = 41 456)		Colon (n = 38 647)		Prostate (n = 6963)	
	Patients, No./total No. (%)	OR (95% CI)	Patients, No./total No. (%)	OR (95% CI)	Patients, No./total No. (%)	OR (95% CI)	Patients, No./total No. (%)	OR (95% CI)	Patients, No./total No. (%)	OR (95% CI)
Insurance status										
Private	15 665/31 251 (50.1)	1 [Reference]	4085/9326 (43.8)	1 [Reference]	3860/12 800 (30.2)	1 [Reference]	4852/14 640 (33.1)	1 [Reference]	476/1735 (27.4)	1 [Reference]
Uninsured	1911/3791 (50.4)	1.06 (0.99-1.13)	584/1083 (53.9)^b^	1.50 (1.32-1.71)^b^	425/1222 (34.8)^b^	1.23 (1.09-1.40)^b^	612/1618 (37.8)^b^	1.22 (1.10-1.36)^b^	74/242 (30.6)	1.09 (0.81-1.48)
Medicaid	5448/11 236 (48.5)	0.95 (0.90-0.99)	1547/3082 (50.2)^b^	1.26 (1.16-1.37)^b^	1075/3097 (34.7)^b^	1.21 (1.11-1.31)^b^	1450/3914 (37.1)^b^	1.16 (1.07-1.25)^b^	178/524 (34)^b^	1.30 (1.05-1.62)^b^
Medicare	27 528/63 930 (43.1)	0.90 (0.87-0.93)	4461/9852 (45.3)	1.05 (0.96-1.15)	6910/23 228 (29.8)	0.98 (0.92-1.04)	5970/17 474 (34.2)	1.01 (0.95-1.08)	1024/4299 (23.8)	0.92 (0.78-1.08)
Other government insurance	934/2119 (44.1)	0.88 (0.8-0.96)	89/224 (39.7)	0.84 (0.64-1.11)	155/530 (29.3)	0.95 (0.78-1.15)	159/484 (32.9)	0.95 (0.78-1.15)	26/103 (25.2)	0.96 (0.60-1.53)
Age, y										
18-34	223/392 (56.9)	1.19 (0.97-1.46)	335/775 (43.2)	0.77 (0.66-0.91)	73/202 (36.1)	1.11 (0.83-1.49)	284/833 (34.1)	0.97 (0.83-1.14)	0	NA
35-49	2745/5082 (54.0)^b^	1.16 (1.09-1.23)^b^	1687/3939 (42.8)	0.80 (0.74-0.86)	639/2255 (28.3)	0.84 (0.76-0.93)	1812/5536 (32.7)	0.92 (0.86-0.99)	46/119 (38.7)^b^	1.57 (1.07-2.31)^b^
50-64	20 080/40 690 (49.4)	1 [Reference]	4153/8715 (47.7)	1 [Reference]	4321/13 789 (31.3)	1 [Reference]	4717/13 632 (34.6)	1 [Reference]	581/2023 (28.7)	1 [Reference]
65-79	23 326/51 746 (45.1)	0.90 (0.86-0.93)	3589/7565 (47.4)	1.02 (0.94-1.11)	5710/18 585 (30.7)	1.02 (0.96-1.09)	4525/12 934 (35.0)	1.05 (0.98-1.13)	802/3123 (25.7)	0.96 (0.81-1.13)
≥80	5779/15 846 (36.5)	0.62 (0.60-0.65)	1141/2868 (39.8)	0.75 (0.67-0.84)	1869/6625 (28.2)	0.91 (0.84-0.98)	1867/5712 (32.7)	0.96 (0.89-1.05)	360/1698 (21.2)	0.75 (0.62-0.91)
Sex										
Female	24 147/52 866 (45.7)	1 [Reference]	10 905/23 862 (45.7)	NA	5788/19 060 (30.4)	1 [Reference]	6273/19 103 (32.8)	1 [Reference]	0	NA
Male	28 006/60 890 (46.0)	1.02 (1.00-1.04)	0	NA	6824/22 396 (30.5)	0.99 (0.95-1.04)	6932/19 544 (35.5)^b^	1.12 (1.08-1.17)^b^	5174/6963 (74.3)	NA
Race										
Black	6644/14 911 (44.6)	0.97 (0.93-1.00)	2069/4206 (49.2)^b^	1.15 (1.07-1.24)^b^	1749/5683 (30.8)	1.00 (0.94-1.07)	2370/6502 (36.5)^b^	1.14 (1.08-1.21)^b^	383/1345 (28.5)	1.09 (0.94-1.26)
White	41973/91 734 (45.8)	1 [Reference]	8129/18 092 (44.9)	1 [Reference]	10114/33 379 (30.3)	1 [Reference]	10 029/29 748 (33.7)	1 [Reference]	1317/5256 (25.1)	1 [Reference]
Other^c^	3203/6341 (50.5)^b^	1.11 (1.05-1.17)^b^	602/1350 (44.6)	0.97 (0.86-1.08)	670/2070 (32.4)	1.05 (0.95-1.16)	699/2079 (33.6)	0.98 (0.89-1.08)	73/300 (24.3)	0.90 (0.68-1.19)
Ethnicity										
Hispanic	2097/4507 (46.5)	0.99 (0.93-1.05)	764/1632 (46.8)	1.02 (0.91-1.13)	951/2828 (33.6)^b^	1.11 (1.02-1.21)^b^	981/2802 (35)	1.04 (0.96-1.13)	127/431 (29.5)	1.19 (0.95-1.50)
Non-Hispanic	49 188/107 278 (45.9)	1 [Reference]	9926/21 747 (45.6)	1 [Reference]	11 448/37 869 (30.2)	1 [Reference]	11 983/35 157 (34.1)	1 [Reference]	1619/6397 (25.3)	1 [Reference]

Abbreviations: NA, not applicable; NSCLC, non–small cell lung cancer; OR, odds ratio.

^a^ The logistic regression models included the sociodemographic variables shown in addition to median income quartile, US Census Division, and year of diagnosis. Results for these variables are available upon request.

^b^ Indicates that the OR is greater than 1 (more likely to present with multiorgan metastases) with a 2-sided *P* < .05. *P* values are available upon request.

^c^ Other includes American Indian, Aleutian, or Eskimo; Chinese; Japanese; Filipino; Hawaiian; Korean; Vietnamese; Laotian; Hmong; Kampuchean (including Khmer and Cambodian); Thai; Asian Indian or Pakistani, no other specification; Asian Indian; Pakistani; Micronesian, no other specification; Chamorran; Guamanian, no other specification; Polynesian, no other specification; Tahitian; Samoan; Tongan; Melanesian, no other specification; Fiji Islander; New Guinean; other Asian, including Asian, no other specification and Oriental, no other specification; Pacific Islander, no other specification; and other.

## Results

Overall, 224 684 patients with stage IV cancer were identified. The median (IQR) age was 66 (58-75) years, 90 664 patients (40.4%) presented with multiorgan metastatic involvement, and 114 891 (51.1%) were female. In total, 32 647 patients (14.5%) were Black, 178 209 (79.3%) were White, and 12 140 (5.4%) were of other races (see the definitions in the Table footnotes).

For all 5 cancer types, uninsured status (time ratio [TR], 0.54; 95% CI, 0.51-0.56; *P* < .001) and, to a lesser extent, Medicaid (TR, 0.69; 95% CI, 0.67-0.72; *P* < .001) and Medicare (TR, 0.71; 95% CI, 0.69-0.73; *P* < .001) enrollment were associated with greater mortality than private insurance (Figure). Multiorgan involvement was associated with a poorer prognosis than single-organ involvement (TR, 0.59; 95% CI, 0.57-0.60; *P* < .001).

**Figure.**
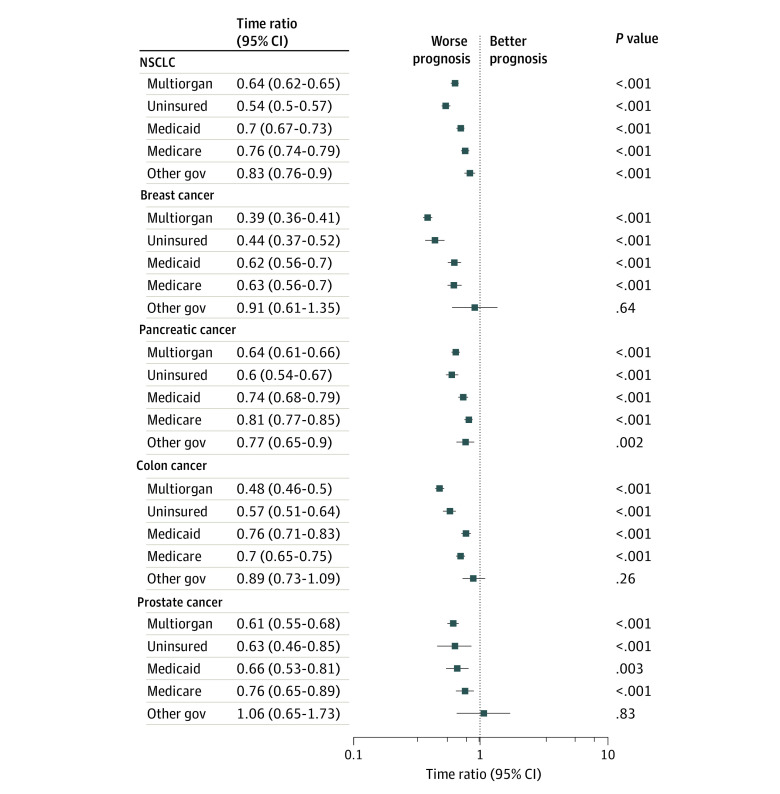
Multivariable Time-to-Event Survival Analysis Evaluating Survival by Insurance Status and Extent of Metastases at Presentation Covariates included the variables listed in the Table as well as median income quartile, US Census Division, year of diagnosis, and multiorgan metastatic presentation (yes/no). The time ratios for payer status and multiorgan metastatic presentation are provided. The reference group for multiorgan metastases was single-organ metastases. The reference group for all insurance groups shown was private insurance. A time ratio of greater than 1 indicates that the variable is associated with longer survival (accelerates survival time). A hypothetical time ratio of 0.5 can be interpreted as the median time to death for patients with that characteristic being half the median time to death for those in the reference group. The 2018 Participant User File was used. Nonregional nodal metastases were considered stage IV for all cancer types. Metastatic involvement of only distant lymph nodes (6011 patients) represented 4.5% of all patients with single-organ metastases (134 020 patients). Patients who had multiple types of cancer in their lifetime were excluded, and 10 722 patients (4.5%) were excluded for missing data regarding sites of metastasis (bone, brain, liver, lung, distant lymph node, or other). A further 394 male patients with breast cancer (1.6% of all patients with breast cancer) were excluded because of small sample size. The sociodemographic characteristics of the patients excluded because of missing data strongly resembled those of the included patients, although some clinically small differences showed significance because of the large sample sizes. A multiple imputations strategy was implemented to account for the 17.4% of patients who had missing values for covariates. Sex was not used for the time-to-event or logistic regression models for breast and prostate cancer. NSCLC indicates non–small cell lung cancer; other gov, other government insurance.

Factors associated with the multiorgan pattern of metastatic involvement were assessed in an attempt to understand the association between payer status and stage IV cancer survival (Table). Uninsured and Medicaid-enrolled patients were more likely to present with multiorgan involvement for all cancers except non–small cell lung cancer. The associations for the other sociodemographic covariates, US Census Division, and year of diagnosis were heterogeneous and tumor specific.

## Discussion

In this cohort study, payer status was associated with stage IV cancer prognosis, with uninsured patients exhibiting the worst survival. These disparities may relate to receiving a diagnosis later in the course of stage IV disease, potentially reflecting decreased access to general practitioners, cancer screening, and treatment.^1,2,3^ Although modest in magnitude, the finding that underinsured patients were more likely to present with multiorgan involvement, which carries a worse prognosis, was observed across multiple cancer types and is consistent with delayed presentation. Further exploration into the social determinants of health is needed,^4^ as the poorer prognosis of socioeconomically disadvantaged patients persisted even after controlling for metastatic burden. Future investigation into the demographics of uninsured and Medicaid-enrolled patients may guide efforts toward earlier intervention.

Other notable socioeconomic disparities were identified. Possibly as a result of unequal implementation of screening and guidelines that are suboptimal for certain populations,^5,6^ disparities existed among cancers with well-established screening guidelines. For example, Black patients with breast and colon cancer were more likely to present with multiorgan involvement. This represents an important domain for future exploration. Limitations included the lack of patient-level income data, as the National Cancer Database abstracts this from zip codes, and the lack of data on insurance status changes over time.

In conclusion, these findings suggest that payer status is associated with survival in stage IV cancer, with uninsured patients faring the worst. Multiorgan metastatic involvement at presentation is associated with a worse prognosis than single-organ involvement, with uninsured and Medicaid-enrolled patients being more likely to present with the multiorgan pattern. Further study into sociodemographic attributes and timeliness of stage IV cancer presentation may expose opportunities to improve cancer outcomes in the US.
